# Detrimental consequences of micropolymers associated plasticizers on endocrinal disruption

**DOI:** 10.1016/j.mtbio.2024.101139

**Published:** 2024-06-24

**Authors:** Utsa Saha, Puja Kumari, Aishee Ghosh, Adrija Sinha, Snehashmita Jena, Apoorv Kirti, Abha Gupta, Anmol Choudhury, Faizan Zareen Simnani, Aditya Nandi, Rudra Narayan Sahoo, Shalini Singh, Richa Mishra, Nagendra Kumar Kaushik, Deobrat Singh, Mrutyunjay Suar, Suresh K. Verma

**Affiliations:** a KIIT School of Biotechnology, KIIT University, Bhubaneswar, 751024, Odisha, India; b Department of Biotechnology, Vinoba Bhave University, Hazaribagh, Jharkhand, 825001, India; c RECETOX, Faculty of Science, Masaryk University, Kotlarska 2, Brno, 61137, Czech Republic; d Markham College of Commerce, Vinoba Bhave University, Hazaribagh, Jharkhand, 825001, India; e Department of Computer Engineering, Parul University, Ta. Waghodia, Vadodara, Gujarat, 391760, India; f Plasma Bioscience Research Center, Department of Electrical and Biological Physics, Kwangwoon University, 01897, Seoul, South Korea; g Condensed Matter Theory Group, Materials Theory Division, Department of Physics and Astronomy, Uppsala University, Box 516, SE-751 20, Uppsala, Sweden

**Keywords:** Microplastic, Nanoplastic, Plasticizers, Hormones, Endocrine disrupting chemical, Hypothalamus-pituitary axis

## Abstract

The prevalence of polymer usage in everyday activities has emerged as a detriment to both human life and the environment. A large number of studies describe severe impacts of micropolymers (MP) and nanopolymers (NP) on various organ systems, including the endocrine system. Additionally, plasticizers utilized as additives have been identified as endocrine-disrupting chemicals (EDCs). MP/NP, along with associated plasticizers, affect principal signalling pathways of endocrine glands such as the pituitary, thyroid, adrenal, and gonads, thereby disrupting hormone function and metabolic processes crucial for maintaining homeostasis, fertility, neural development, and fetal growth. This review delves into the sources, distribution, and effects of micropolymers, nanopolymers, and associated plasticizers acting as EDCs. Furthermore, it provides a detailed review of the mechanisms underlying endocrine disruption in relation to different types of MP/NP.

## Highlights

• Microplastics and nanoplastics are rigoursly increasing in ecosystem day by day.
• The hazardous effects of micro/nanoplastics has been important concern on endocrinal system disruption.
• Released micro/nanoplastics does a mechanistic association with endocrinal disruption chemicals.
• Mechanistic details of endocrinal disruption pathway due to micro/nanoplastics has been detailed.

## Graphical abstract

**Figure:**
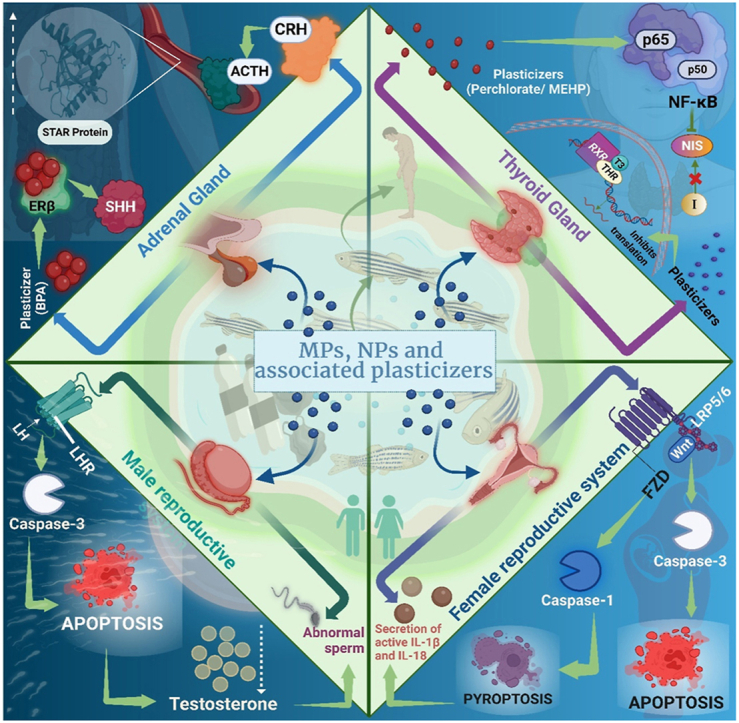

## Abbreviations

MP: Micropolymer
NP: Nanopolymer
EDC: Endocrine Disrupting Chemical
PBDE: Polybrominated diphenyl ethers
MEHP: Mono(2-ethylhexyl) phthalate
DEHP: di(2- ethylhexyl) phthalate
PVC: Polyvinyl chloride
PCB: Polychlorinated biphenyl
PCC: Polybrominated biphenyl
PS-MP: Polystyrene microplastic
PS-NP: Polystyrene nanoplastic
BPA: Bisphenol A
APE: Alkylphenol ethoxylate
NoP: Nonylphenol
OH-BDEs: Hydroxylated polybrominated diphenyl ethers
BDE3: 4-Bromophenyl ether
TH: Thyroid hormone
TDC: Thyroid disrupting chemical
T3: Triiodothyronine
T4: Thyroxin
TSH: Thyroid stimulating hormone
TTR: Transthyretin
HPT: Hypothalamic-pituitary thyroid
SSH: Sonic Hedgehog
HPA: Hypothalamus-pituitary adrenal
IPEN: International Pollutants Elimination Network

## 1 Introduction

Plastic is an indispensable part of modern life, ubiquitous in a vast array of commercial products. In 1907, Leo Baekeland revolutionized the material world by introducing Bakelite, the first fully synthetic plastic [1]. The widespread use of plastics is attributed to their versatility, lightweight nature, durability, flexibility, and cost-effective production. Plastics can be moulded, extruded, or pressed into solid objects of diverse shapes owing to their fluidity. Most contemporary plastics are derived from fossil fuels, such as natural gas or petroleum. These can undergo various chemical and physical degradation processes, including biodegradation, photodegradation, and hydrolysis, and breaking down into micro- and nanoscale particles [2]. Through primary and secondary fragmentation, these micropolymers (MPs) further degrade into billions of nanopolymers (NPs), facilitating their global distribution [3]. However, due to their inherent durability, these polymers persist in the environment for extended periods, causing substantial ecological harm (see Table 1, Table 2, Table 3, Table 4, Table 5).

**Table 1:** Distribution of micropolymers (MPs) and nanopolymers (NPs).

Distribution	Size of MPs	Method of sampling	Reference
Global	<1 mm	Whole water filtration	[4]
Global	0.33–4.75 mm	Plankton nets (0.33 mm)	[5]
Yangtze Estuary, China	0.5–5 mm	Plankton nets (0.333 mm)	[6]
North-Eastern Pacific ocean	0.333–5 mm	Whole water collection using sieves (62.5 μm)	[7]
Commercial molluscs in Northern Tunisia	1.1–1 mm	–	[8]
Face scrubs	<100 nm	Sequential filtration	[3]
Europe and Arctic	11–150 μm	Wet deposition of the snow	[9]
Baltic sea	10–800 μm	Wet deposition	[10]

**Table 2:** Source, mechanism of entry, and effects of exposure to MP/NP and associated plasticizers.

Exposure route	Source of MP/NP exposure	Mechanism of Entry	Effects	References
Gastric	Ingestion of contaminated food/drink	- Infiltration of Peyer's patches via phagocytosis or endocytosis. -Adsorption or persorption through single layer of intestinal epithelium.	Pro-inflammatory responses, morphological changes in and inhibitory responses in gastric adenocarcinoma cells.	[11,12,[13], [14], [15]].
Pulmonary	Inhalation of contaminated aerosols	Penetration through the capillary system into the bloodstream, leads to whole-body circulation.	- Chronic inflammation due to the release of chemotactic factors. - Prevention of macrophage migration.	[11,12,14,16,17]
Dermal	Contact with beauty products or environmental pollutants containing MPs/NPs.	- Penetration through stratum corneum, - Entry through sweat glands, skin wounds, and hair follicles.	- Disruption of tight-junction protein expression.	[14,[18], [19], [20]]

**Table 3:** Applications and the route of exposure of various plasticizer compounds leading to adverse health effects.

Plasticizer compounds	Applications	Exposure route	Health effects	References
Phthalates	- Excipients in certain medications. - Scent retainers **-** Children's toys.	- Leaching into the environment, indoor air. - From packaging materials into food and beverages.	- Endocrine disruption. - ADHD in kids and poorer sperm quality in men.	[[21], [22], [23],^24^,^25^,^26^]
Bisphenol A (BPA)	Plasticizers for polycarbonate plastics and epoxy resins are used in food containers, dental implants, water pipes, toys etc.	- Leaching from polycarbonate plastics and epoxy resins into food, and beverages. - Hydrolysis of BPA under high temperature or pH.	- Endocrine disruption. - Linked to cancer, cardiovascular diseases, and disruptions in neuroendocrine and reproductive systems.	[27,28,29]
Alkylphenol Ethoxylates (APEs)	Used in emulsifiers, detergents, solubilizers, antistatic agents, and dispersing agents in various domestic, industrial, and agricultural products.	-Biodegradation into more toxic substances. **-** Ingestion of contaminated food and drinking water. **-** Dermal absorption.	Degraded APEs produce toxic compounds which have estrogenic and anti-androgenic effects.	[[30], [31], [32], [33],^34^,^35^]
PBDEs	Flame retardants in furniture, textiles, electric and electronic devices.	Leaching from products during use, disposal or recycling.	- Interference with thyroid hormone signalling. **-** Endocrine disruptor.	[[36], [37], [38], [39], [40], [41]].
Organotin	Biocides, wood preservatives, antifouling paints, agricultural fungicides.	Transfer through contact, contaminated dietary sources.	Endocrine disruption.	[42,43,44,45].

**Table 4:** Importance of the hypothalamus, pituitary gland and the different hypothalamus-pituitary axes, focusing on the disruptive effects of EDCs on their functioning.

Aspect	Importance	Effect of EDCs	Effects on human	References
Hypothalamus	Regulates various physiological processes by secreting hormones that influence pituitary activities.	Activates toll-like receptors leading to inflammatory responses, suppresses acetylcholinesterase function and alters neurotransmitter levels.	Disruption in signalling pathways, affecting growth, lactation, metabolism, and milk supply.	[46,47]
Pituitary	Releases hormones under the influence of hypothalamic tropic hormones.	Alters pituitary hormone activation (e.g., prolactin, TSH).	Delayed formation of secondary sexual characteristics, infertility, stunted fetal development.	[48,49].
Hypothalamus-Pituitary-Thyroid Axis (HPT)	Regulates thyroid function by controlling TSH levels.	Significantly alters thyroid stimulating hormone levels which affects the homeostasis and the glucose levels.	Homeostasis disruption, glucose level alteration, thyroid function impairment.	[50,[51], [52], [53], [54]]
Hypothalamus-Pituitary-Adrenal Axis (HPA)	Regulates stress response and metabolism.	Affects hormonal secretions from the cortex and medulla regions.	Impaired stress response, Lowers adrenal gland weight.	[[55], [56], [57], [58]]
Hypothalamus-Pituitary-Gonadal Axis (HPG)	Regulates reproductive functions in both males and females.	Decreases kisspeptin levels in zebrafish, alters GnRH levels, and disrupts FSH and LH ratio.	Male and female infertility, stunted fetal development, disruption of secondary sexual characteristics.	[59,60]

**Table 5:** Summary of action mechanism of MP/NP and associated plasticizers as EDCs, and their effect on the endocrine system of animals and humans.

MP/NP and associated plasticizers	Hypothalamus- pituitary axes affected	Mechanism of action	Effect on animals	Effect on humans	References
Polybrominated Diphenyl Ethers (PBDEs)	Hypothalamus-Pituitary-Thyroid Axis (HPT)	-Alters T4 binding and decreases T4 quantity in serum. -Interferes with gene transcription (e.g., thyroid stimulating hormone subunit, deiodinase type 2)	Alters gene expression related to HPT axis in zebrafish.	-Disrupts human thyroid function by interfering with the transport of thyroid hormones. -Linked to thyroid carcinoma.	[[52], [53], [54],^61^,^62^,[63], [64], [65]].
	Hypothalamus-Pituitary-Adrenal Axis (HPA)	-Rise in Aldosterone and corticosterone levels in the serum. -Upregulates Cyp11b1 expression and disrupts AMP-activated protein kinase (AMPK) signalling.	Exposure in rats increases aldosterone and corticosterone levels and disrupts AMPK signalling.	Further research is needed for direct human impact.	[57]
Bisphenol-A (BPA)	HPT	-Interferes with thyroid hormone (T3) receptor attachment. - Decreases transcriptional activity mediated by thyroid hormone receptors.	In zebrafish, BPA controls the transcription of thyroid-specific enzymes.	- Impacts thyroid hormone action which impacts growth and development. - Affects the follicular cells of the thyroid.	[66,67]
	HPA	- Alters signalling via endoplasmic reticulum and Sonic Hedgehog (SHH) pathway, activating cyclin D1 and cyclin D2, and changing adrenal cell proliferation. - Increases SHH gene transcription, leading to cell proliferation.	Prenatal exposure in mice leads to increased plasma corticosterone levels and StAR protein levels. **-** BPA increased the weight of the adrenal glands in mice.	Higher concentration of BPA in serum of patients with nonfunctional adrenal incidentaloma.	[55,68,69]
	HPG	-Estrogen receptor agonist leading to disruption of hormonal balance.	–	Reproductive tract abnormalities.	[70,71]
Polystyrene Nanopolymers (PS-NPs)	HPT	-Suppresses production of T3, FT3, FT4, and thyroid hormone levels in blood.	- Impairs thyroid functioning in rats.	- Research required	[72]
	HPA	Modifies glucose homeostasis by boosting cortisol secretion; impacts key molecules like neurotransmitters, enzymes, and receptors, affecting brain electrical impulses and behaviour.	Altered glucose homeostasis in zebrafish.	Effects on the human system require further investigation.	[73],
	HPG	- Tight junction damage in the testis. - Deformities in sperm cells with a decrease in motility of sperms. - enter the ovarian granulosa cells and decrease the anti-Müllerian hormone level.	-Decrease in the embryogenesis in oysters. - In earthworms, it damages the sperm's plasma membrane, reducing density of sperm, viability and mature bundle.	Decreased motivation for reproduction, ovarian and uterine fibrosis.	[74,75,[76], [77], [78]].
Phthalates	HPT	- Disrupts thyroid hormone related gene expression (e.g., dio1, dio2, ttr, NK2 homeobox etc.). - Decreases T3 and T4 level.	- Inflation of swim bladder in fish (e.g., Japanese medaka, zebrafish).	- Alters serum TSH and T3 levels in children. - Correlated with thyroid-related issues in females	[79,80,81,82].
	HPG	Reduction of anogenital distance and an incomplete testicular descent.	**-**	Male infertility	[71]
Di-2-ethylhexyl phthalate (DEHP)	HPT	- Induces NF-κB pathway leading to NIS down-regulation	- Not specified	- Down-regulation of thyroid iodine uptake - Altered blood TSH and T3 levels.	[51,83,84],
	HPA	Linked to decreased angiotensin II expression.	–	- Lowers the aldosterone level in adult adrenal glands.	[56].
	HPG	- Inhibition of testosterone synthesis. - Altes the genes responsible for reproductive tract development.	Testicular abnormalities, decreased sperm quality, and altered hormone levels in rodents.	-Delayed formation of secondary sexual characteristics, male infertility. - A birth weight reduction of the fetus. - Decrease in anogenital distance in both males and females.	[85,71,77].
Phenols	HPA	Disrupts the adrenal gland's endogenous estrogenic cascade.	Results in the absence of the HPA axis' negative feedback loop in *Podarcis sicula* lizard.	- Disruption of adrenal functioning.	[86],
	HPG	Estrogen receptor agonist.	**-**	Male infertility, developmental issues	[71]
Microplastics (MPs)	HPT	Reduction of Thyroid Hormone's metabolic capacity.	**-**	Decreased control of metabolism, growth, reproduction, and development.	[87]
	HPG	- MPs get internalized by Leydig cells. - Downregulation of the LH-mediated LHR/cAMP/PKA/StAR pathway. - Trigger the TR4/NOX2 signalling axis inducing oxidative stress	- Abscised and disorderly arranged spermatogenic cells and multinucleated gonocytes in the seminiferous tubule in mice. -Decreased motivation for reproduction in Daphnia magna.	- uterine fibrosis.	[75,[88], [89], [90],^91^]

Advancements in plastics production have incorporated numerous additives, such as flame retardants, stabilizers, colourants, and plasticizers [[92], [93], [94]]. Despite these enhancements, most plastics are not recycled, ultimately ending up in landfills, water bodies and the atmosphere, thus contributing to environmental pollution. MPs and NPs have the potential to bind with persistent organic pollutants (POPs), serving as carriers of these harmful substances into human systems [95]. The endocrine system, like various other organ systems, is vulnerable to the detrimental effects of MPs and NPs. This system comprises hormone feedback loops and an intricate network of glands and organs, regulating metabolism, energy levels, reproduction, growth, development, and responses to injury, stress, and mood. The term “hormone” is occasionally expanded to cover substances made by cells that influence other cells nearby (paracrine signalling) or within the same cell (autocrine or intracrine signalling). Hormones produced by glandular cells travel through the circulatory system to target distant organs, influencing physiology and behaviour [96]. Key glands in the endocrine system include the hypothalamus, pituitary, thyroid, thymus, adrenal glands, pancreas, ovaries, and testes.

This review delves into the global distribution of MPs, NPs, and plasticizer additives. It examines the exposure pathways of MPs and NPs to the human body and identifies the primary endocrine glands affected by these pollutants. The review emphasizes on different studies that highlight the endocrine-disrupting properties of MPs, NPs, and plasticizers. Finally, it addresses existing research gaps to foster a better understanding of preventive measures necessary to mitigate the issue of endocrine disruption by MPs, NPs and associated plasticizers.

## 2 Micro/nanoplastic distribution

The worldwide havoc caused by MPs/NPs is raising serious concern for the health of this planet. Although the exact percentage of plastic content prevailing currently is still controversial, MPs/NPs have been distributed to all types of ecosystems being it be aquatic or terrestrial [97]. China (26 %), Europe (20 %), and North America (19 %) are the top three global producers of plastic [2]. The amount of MPs/NPs that could be transported via the marine atmosphere and dumped into the oceans annually ranges from 0.013 to 25 million metric tonnes [97]. According to a UN report, over 800 species of life have been affected by plastic waste through ingestion or entanglement of MP/NP from the environment. These numbers have exceeded 69 % of the data recorded in 1977, where only 247 species were found to be affected [98]. Such impacts have been related to the distribution of MPs/NPs through abandoned fishing gear, effluent discharge, tourism-related activities, and microfibre release from washing clothes in aquatic habitats [[99], [100], [101]]. Seas all around the world will contain more plastic than fish by the year 2050, according to a 2016 report by the Ellen MacArthur Foundation, which was also covered by several international publications [[102], [103], [104]]. Even the most distant habitats, including polar regions and highland lakes, can harbour MPs and NPs [[105], [106], [107], [108]].

These plastics being widespread in the environment is evident to interact with living organisms [109]. MPs/NPs can be adsorbed on the surface of plants like some vascular plants and NPs have also been observed to get internalized by plants, thus increasing their concentration in the kingdom plantae. Plastics can have a variety of phytotoxic effects on vascular plants, which include negative effects on growth, oxidative stress, and photosynthesis [110]. MPs gather on seed capsule pores and prevent terrestrial vascular plants from germinating and growing roots. NPs show higher toxicity as compared to the effects of MPs [111]. MPs/NPs distribution to the animal world is also under huge consideration, especially those animals who share a close interaction with humans. These plastics accumulated in the gut of shrimps and crustaceans, hampering their gut microbiota and increasing tissue damage and oxidative stress [112]. Major aquatic animals reported for ingestion of MP/NP consists of fishes, turtles, seabirds, worms, and crustaceans [11,113]. They affect the general physiological properties by bioaccumulation in various important organ systems, hampering their reproduction as well. In consequence, MPs/NPs are also responsible for the decreasing population of various species which is concerning for ecological balance.

The availability of MPs/NPs in various animals and plant species is ingested by humans due to biomagnification through the food chain. On the contrary, these are not the only sources of MPs/NPs. Several food items that are part of daily intake have been reported to contain such plastic particles too. Fruits and vegetables contain MPs, with a higher concentration in fruits [114]. They are also reported to be found in table salt and drinking water [115]. Friction in tires, burning of plastic waste, industrial smoke, etc., serve as major pollutants for the circulation of MPs/NPs in the atmosphere. Some of the MPs/NPs remain suspended in the atmosphere and, upon inhalation, enter the respiratory system [116].

Extensive studies have been carried out to investigate the presence of MPs/NPs in various organisms, including plants, animals, food, and drinking water. However, to the best of our knowledge, there has been limited research conducted to firmly establish this point on a statistical basis. It is crucial to not only understand the distribution of MPs/NPs but also to explore the mechanisms for their identification, along with conducting thorough toxicological analyses. Further investigation is needed to substantiate any concrete claims in this area. The table lists a few sources of MP/NP pollutants.

## 3 Exposure to MPs/NPs: from the environment to the human body

The journey of microplastics and nanoparticles (MPs/NPs) from the environment to the human body is a topic of growing concern. Understanding the mechanisms and pathways through which these particles enter our bodies is essential for assessing the potential risks they pose to human health. Various exposure pathways to humans include activities like agriculture, aquaculture, fishing, industrial activities, and products like urban dust, tyres, cosmetics, synthetic textiles, wastewater treatment plants, large plastic fragmentation, runoff, and solid waste management [111,[9], [10], [12], [117]]. The distribution of MPs and NPs in water bodies are one of the important aspects of their distribution in environment. Consequently, High-density MPs/NPs sink to the deepest sediment layers, while low-density MPs/NPs remain on the surface of water bodies such as oceans, rivers, and seas. Marine organisms’ intake these MPs/NPs present at different water layers, which also lead to enter these particles in food chain through trophic transfer via seafood [9,13].

Research has shown that MPs/NPs can enter the human body through various routes [9,10]. Inhalation is one of the primary pathways, as these particles can be released into the air through industrial processes, vehicle emissions, and the degradation of plastic waste. Once airborne, they can be easily inhaled and transported deep into the respiratory system. Another significant route of exposure is through the consumption of contaminated food and water. MPs/NPs can accumulate in aquatic environments, where they are ingested by marine organisms. When humans consume seafood or water contaminated with these particles, they can unknowingly introduce MPs/NPs into their bodies. Furthermore, dermal exposure is another pathway through which MPs/NPs can enter the human body. Personal care products, such as cosmetics and skincare items, often contain microplastics. When applied to the skin, these particles can be absorbed into the body. Once inside the body, MPs/NPs can migrate to different organs and tissues. Studies have shown that they can penetrate the gastrointestinal tract, reach the bloodstream, and even cross the blood-brain barrier. The long-term effects of this accumulation and distribution within the body are still not fully understood, highlighting the need for further research.

Hence, the exposure of humans to MPs/NPs encompasses various routes, including inhalation, ingestion, and dermal contact. Understanding the mechanisms and potential health impacts of this exposure is crucial for developing effective mitigation strategies and safeguarding human well-being.

### 3.1 Gastric exposure to MP/NP

The most prevalent way a human gets exposed to MP/NP is via ingestion [11,12]. Studies have shown the presence of MP/NP in the excreta of humans, which suggests that MP/NP are being consumed by humans through food or drink. Other researches show the uptake of MP/NP in the environmental models which proves that humans have been ingesting MP/NP regularly [11,117]. Domenech et al. reported some studies related to different consumer items and the amount of MP/NP present in them such as fruits and vegetables, seafoods, drinking water, plastic packaging of foods, table salt sugar, alcohol, and cereals [[13], [14], [15]]. MP/NP enter via lymphatic tissue in the Gastrointestinal (GI) tract. MP/NP infiltrates the microfold cells present in the Peyer's patches through phagocytosis or endocytosis [118]. The Adsorption and persorption are other probable mechanisms through which MP/NP internalize in the cells. Through this mechanism, MP/NP travel paracellularly through the single layer of the intestinal epithelium [14]. Pro-inflammatory responses, morphological changes and inhibitory responses were seen in human gastric adenocarcinoma cells [14]. It is important to note that the long-term health effects of gastric exposure to MPs/NPs are not yet fully understood, and more research is needed to assess the risks comprehensively. Regulatory measures and initiatives to reduce plastic pollution and limit the presence of MPs/NPs in food and beverages are being implemented to mitigate these potential risks.

### 3.2 Pulmonary exposure to MPs/NPs

Pulmonary exposure to microplastics and nanoparticles (MPs/NPs) occurs when these particles are inhaled and enter the respiratory system. This route of exposure is of concern as it directly introduces MPs/NPs into the lungs, potentially leading to adverse health effects. MPs/NPs can be present in the air we breathe due to various sources, including the breakdown of larger plastic particles, industrial emissions, and atmospheric deposition. These particles can range in size from nanometers to micrometers, allowing them to reach different parts of the respiratory system. It is estimated that a person inhales 26–130 particles of MPs/NPs per day [14,16]. Humans can get exposed to MP/NP through breathing, contaminated aerosols from ocean waves, particles of airborne fertilizers and dried wastewater treatments. Due to the vast surface area of the lungs and fine tissue barrier of less than 1 μm, these MPs/NPs can traverse through the capillary system and enter the bloodstream. This results in the whole-body circulation of MP/NP to all organs and cells [12,118]. After reaching the lungs MP/NP can result in chronic inflammation due to the severe release of chemotactic factors which prevents macrophage migration, known as dust overload [118,17]. There are concerns about the potential for MPs/NPs to translocate from the lungs to other organs, such as the bloodstream, lymphatic system, or even the brain. This translocation could lead to systemic effects and impact overall health.

### 3.3 Dermal exposure to MPs/NPs

MP/NP can be exposed to the body through dermal contact from sources like beauty products (scrubs, body wash, body lotion, moisturizer, face wash, etc.) [119]. The dermal route might be chosen by the monomers and additives of plastics e.g., endocrine disruptors like Bisphenol A (BPA) and phthalates [14]. Absorption of MPs/NPs occurs through the stratum corneum of skin owing to their hydrophobic nature, although they can gain entry through sweat glands, kin wounds or hair follicles [118,18]. Polystyrene particles ranging from diameters 20–200 nm bear the capability to penetrate through the stratum corneum [118,19]. Intercellular adhesion in skin exposed to UV radiation gets thwarted due to the truncated expression of tight-junction proteins Zonula occludens-1, and claudin-1, thus increasing penetration of MP/NP in the skin [20].

## 4 Plasticizers as a menace to biotic health

### 4.1 Phthalates

Phthalates are synthetic chemicals commonly called as diesters of phthalic acid (1,2-benzene dicarboxylic acid) and are widely used in various consumer and industrial products due to their versatility [120]. They primarily function as plasticizers in plastics, which are used in products such as children's toys, medical formulations, and personal care items to enhance flexibility and durability [21,22]. Additionally, they serve as scent carriers in certain cosmetics and toiletries. Despite their usefulness, phthalates are known for their weak bonds with plastic polymers, making them prone to leaching out into the environment. This leaching can occur from products into indoor air, house dust, and even food and beverages stored in plastic containers, leading to human exposure [[24], [25], [121]]. Exposure to phthalates has been linked to numerous health concerns [24]. Even low levels of phthalate metabolites in the body have been associated with adverse effects [25,122]. For instance, studies have suggested connections between phthalate exposure and conditions like ADHD in children and reduced sperm quality in men [26]. These health effects are particularly concerning because phthalates are pervasive in indoor environments where people spend a significant amount of their time. Given these risks, organizations such as the Endocrine Society and the International Pollutants Elimination Network (IPEN) have highlighted the broader health impacts of phthalates and other endocrine-disrupting chemicals found in plastics. Their cumulative efforts underscore the importance of minimizing human exposure to these substances through regulatory measures and consumer awareness.

### 4.2 Bisphenol A

Bisphenol A (BPA), chemically known as 2,2-bis(4-hydroxyphenyl) propane, is one of the most heavily produced chemicals globally. It serves a multitude of purposes in industry and consumer goods. Polycarbonate plastics and epoxy resins, which are widely used in various products, particularly benefit from BPA due to its properties as a key building block. BPA can be found in everyday items such as food containers (including plastic bottles and linings of canned goods), thermal paper receipts, water pipes, toys, dental sealants, medical equipment, and electronics [[27], [123], [124], [125]]. Its versatility and durability make it a preferred material in these applications. Despite its widespread use, concerns have been raised about the health implications of BPA exposure. Humans are exposed to BPA through both dietary sources (like food and beverages stored in BPA-containing containers) and non-dietary sources (such as handling thermal paper receipts). BPA can leach from these products, leading to human exposure through ingestion, inhalation, or skin contact. Research has linked BPA exposure to potential adverse health effects, especially in vulnerable populations such as pregnant women, foetuses, and young children [126]. Some studies suggest that BPA may act as an endocrine disruptor, potentially interfering with hormonal systems in the body. This has raised concerns about its possible role in various health conditions, although the extent and exact mechanisms of these effects continue to be studied and debated.

BPA is commonly selected as a plasticizer for its effective cross-linking properties. However, during the polymerization process, unbound monomers may persist and subsequently be released into the environment [127,128]. BPA molecules are bound together by ester bonds, which are susceptible to hydrolysis when exposed to elevated temperatures or acidic or basic substances [28]. Studies have demonstrated that BPA can leach from polycarbonate plastics, epoxy resins, and other items in contact with food and beverages. It has recently been demonstrated that BPA may cause mutagenesis and cancer in animal studies. As an endocrine disruptor, BPA harms various tissues and organs, such as the immune system, reproductive system, and neuroendocrine system [29]. BPA, as an estrogenic endocrine disrupting chemical, exerts developmental effects on adipocytes, leading to potential implications for adult obesity and impaired reproductive capacity, highlighting its multifaceted role in disrupting endocrine function and metabolic homeostasis [129]. The Consortium Linking Academic and Regulatory Insights on BPA Toxicity (CLARITY-BPA) program was established through collaboration between the National Institute of Environmental Health Sciences (NIEHS), the National Centre for Toxicological Research (NTP), and the Food and Drug Administration (FDA) to comprehensively investigate the potential health impacts of BPA exposure. It not only assessed BPA toxicity in rodents but also provided animals and tissues, subjected to controlled BPA doses, for use in the studies. The study maintained strict controls over variables such as the strain and number of animals, diet, housing conditions, BPA doses, and the route of BPA exposure. The CLARITY-BPA study found few significant effects of BPA treatment overall, with some minor weight and pathology changes deemed of questionable relevance. Notably, an increase in mammary gland adenocarcinoma incidence was observed in female rats at a low BPA stop-dose but not at continuous doses, raising questions about biological plausibility. The reference estrogen (EE2) group exhibited multiple significant effects, particularly in female reproductive tissues and mammary glands, underscoring its potency as an endocrine disruptor [130]. Since BPA crosses the human placental barrier and appears in the serum of fetuses during gestation, researchers hypothesized that BPA negatively impacts neurodevelopment, potentially leading to behavioural disorders later in life [131]. However, the precise mechanisms by which BPA induces its specific cellular and tissue effects on neurodevelopmental processes are still not well understood at the mechanistic level and require further research.

Given its ubiquitous presence in consumer products and potential health risks, there have been efforts to regulate and reduce BPA exposure. Many manufacturers have started to produce BPA-free alternatives in response to consumer demand and regulatory measures aimed at minimizing exposure levels. Overall, while BPA offers significant benefits in manufacturing and product performance, ongoing research and regulatory scrutiny are essential to better understand and mitigate potential health risks associated with its use.

### 4.3 Alkylphenol ethoxylates (APEs)

Alkylphenol ethoxylates (APEs) are a group of non-ionic surfactants widely used in various industrial, agricultural, and domestic products due to their emulsifying, detergent, solubilizing, antistatic, and dispersing properties. These chemicals are also found in plastic additives and pesticides, contributing to their broad application [[30], [31], [32]]. In the environment, APEs undergo biodegradation processes that lead to the formation of alkylphenols (APs) such as nonylphenol (NoP) and octylphenol (OP) [33]. These APs are considered more toxic than their ethoxylated counterparts. Exposure to APs primarily occurs through the ingestion of contaminated food and drinking water, though dermal absorption and inhalation are additional potential pathways [31,[34], [132], [133]].

Nonylphenol, in particular, is semi-volatile and can enter the atmosphere, returning to surface water ecosystems through precipitation [134]. It exhibits significant estrogenic effects by mimicking the natural hormone 17β-estradiol, albeit with lower affinity for hormone receptors [132,135]. This estrogenic activity can disrupt endocrine functions in organisms exposed to NoP. Furthermore, nonylphenol exerts anti-androgenic effects by interfering with androgen receptor activation, which is crucial for normal male reproductive system development. This disruption occurs at multiple stages of androgen receptor signalling [35]. Due to the known harmful effects of nonylphenol and other degradation products from APEs, including their endocrine-disrupting properties, regulatory measures have been implemented in various regions. In the European Union, for instance, the use and production of APEs, especially those leading to the formation of nonylphenol, have been restricted or prohibited to mitigate environmental and health risks [132]. Overall, while APEs serve important industrial functions, their potential to degrade into more harmful substances like nonylphenol underscores the importance of responsible usage and regulatory oversight to protect ecosystems and human health.

### 4.4 Polybrominated diphenyl ethers (PBDE)

Polybrominated diphenyl ethers (PBDEs) are synthetic flame retardants widely employed in consumer products such as furniture, textiles, electronics, and other goods to reduce the risk of fire. Due to their extensive use, PBDEs have been produced and incorporated into various materials [36,37]. One of the significant concerns with PBDEs is their tendency to leach out from products due to their non-covalent attachment to polymer constituents during manufacturing, use, disposal, and recycling processes. This leaching can result in environmental contamination and human exposure through multiple pathways.

PBDEs are classified as endocrine-disrupting chemicals (EDCs) because they can interfere with hormone systems in the body [38,39]. Structurally, PBDEs resemble thyroid hormones, which are crucial for regulating metabolism and development. This structural similarity allows PBDEs to mimic thyroid hormone activity, leading to disruption in thyroid hormone signalling pathways [40]. The interference with thyroid hormone signalling by PBDEs can have serious consequences for health. Thyroid hormones play vital roles in metabolism, growth, and brain development, particularly in foetuses and infants. Disruption of these processes by PBDEs can potentially lead to developmental delays, neurobehavioral disorders, and other health problems. Due to their persistence, bioaccumulation potential, and harmful effects on health [41], several PBDE congeners have been restricted or banned in various countries. Efforts to phase out the use of PBDEs have led to the development of alternative flame retardants that are considered less hazardous to human health and the environment.

While PBDEs have provided fire safety benefits in consumer products, their potential adverse health effects, particularly as endocrine disruptors mimicking thyroid hormones, highlight the importance of regulatory measures and the development of safer alternatives to protect public health and the environment.

### 4.5 Organotin

Organotin compounds, such as tributyltin (TBT) and triphenyltin (TPT), have been widely utilized in various industrial and agricultural applications. They serve as biocides, fungicides, wood preservatives, and disinfecting agents in industrial cooling waters. Additionally, they are notorious for their use in antifouling paints applied to marine vessels to prevent the growth of marine organisms like barnacles and algae [42,43]. Despite their effectiveness in these applications, organotin compounds pose significant environmental and health risks. They are persistent pollutants that can accumulate in marine and freshwater ecosystems, often exceeding toxicity thresholds for both acute and chronic exposure [136]. This contamination has raised substantial concerns about their impact on terrestrial and aquatic environments.

Human exposure to organotin primarily occurs through contaminated dietary sources, especially seafood, drinking water, and food stored in cans [44]. Mono- and diorganotins are also used as stabilizers in the production of polyolefin plastics like polyvinyl chloride (PVC). This usage raises concerns about potential transfer of organotin to drinking water and food items through contact [137]. Organotin are classified as endocrine-disrupting chemicals (EDCs) because they interfere with the normal functioning of the endocrine system in various species. Studies have shown that organotin can disrupt hormone signalling pathways, leading to adverse effects on reproductive and developmental processes in vertebrates. For instance, TBT has been particularly implicated in causing masculinization of female marine organisms, which disrupts normal reproductive functions [45]. Due to their environmental persistence and toxicity, regulatory actions have been taken globally to restrict or ban the use of certain organotin, particularly TBT, in antifouling paints and other applications. These measures aim to reduce environmental contamination and protect ecosystems and human health from the adverse effects of organotin exposure. In conclusion, while organotin compounds have had industrial benefits, their environmental persistence and potential health impacts, particularly as EDCs, highlight the importance of continued monitoring, regulatory oversight, and the development of safer alternatives in industrial and consumer applications.

## 5 Mechanism of interaction between MPs and various endocrine disrupting chemicals

**Fig. 1:**
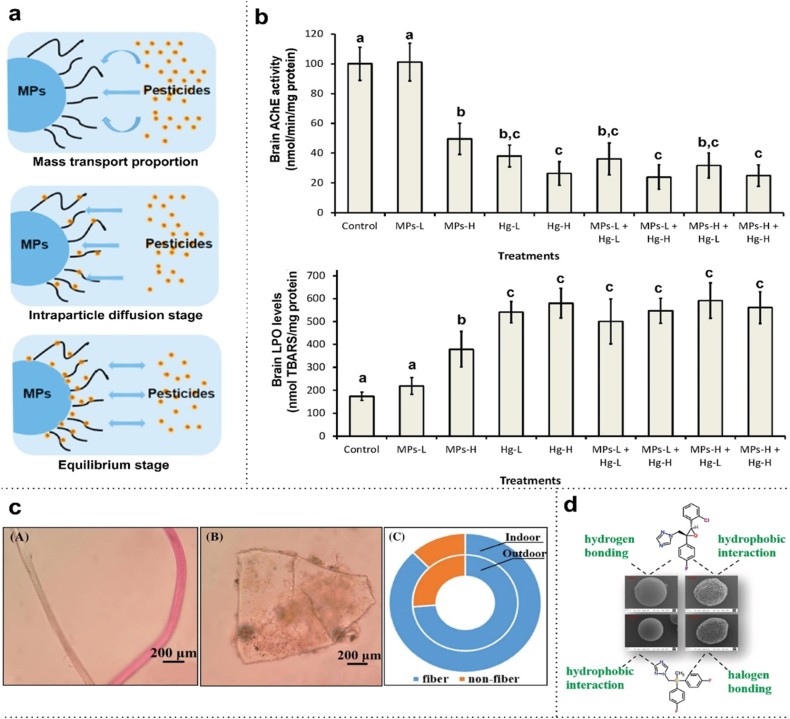
**(a)** Three adsorption steps showing the interaction between micropolymers and pesticides. The wavy lines depict the portion of protrusion on the MP surface [148]. **(b)** Impacts of micropolymers and mercury on neurotoxicity, for instance AChE activity and LPO levels of the brain in *Dicentrarchus labrax* [147]. **(c)** Micrograph images of MPs in the form of (A) fibres (B) granules (C) distribution proportion of MPs found in outdoor dusts (inside cycle) and indoor dusts (outside cycle) [145]. **(d)** Adsorption of azole fungicides on micropolymers [142].

Endocrine disrupting chemicals (EDCs) comprise groups of heterogeneous molecules, including synthetic chemicals. Industrial solvents or lubricants, byproducts like polychlorinated biphenyls (PCBs), polybrominated biphenyls (PBBs), dioxins, fungicides, pesticides, plastics like BPA and phthalates, and pharmaceutical agents like diethylstilbestrol (DES) are examples of EDCs. These disruptors also include natural substances like phytoestrogens, such as genistein and coumestrol, present in both human and animal meals [138]. MPs/NPs, as endocrine-disrupting chemicals from the environment, enter the human body through various sources like packaged foods, drinking water, or personal cosmetics, but can also enter the system by conjugating with other EDCs. Due to their high capacity for adsorption and ability to carry toxins over long distances, MPs are thought to be potential carriers of a variety of contaminants. Researchers have observed that hydrophobic interactions primarily drive sorption mechanisms, particularly for organic pollutants. However, electrostatic forces, van der Waals forces, hydrogen bonding, and pi-pi interactions also play significant roles [139]. Pesticides used for various agricultural purposes act as EDCs which also bind with MPs increasing their adsorption properties, and these conjugate exhibits various toxic effects [140]. MPs like polyethylene and polypropylene show adsorption properties on pesticides like carbofuran and carbendazim, which are highly toxic to humans. The adsorption also showed a negative correlation with the size of MPs, indicating a relation with surface area. The adsorption process between MPs and pesticides occurs in three steps – mass transport proportion, intraparticle diffusion stage, and equilibrium stage (Fig. 1a) Properties of MPs, such as the size of the individual particle, bear a relationship with the adsorption capacity on the surface of fungicides. The bonding capacities are inversely proportional to each other, i.e., the smaller the size of the particle, the greater the tendency to get adsorbed. Azole pesticides get adsorbed on MPs via bonds hydrogen bonding, hydrophobic interaction, and halogen bonding (Fig. 1d) [141,142]. Electrostatic interactions and surface complexation are the principal mechanisms by which heavy metal ions like those of Pb, Cd, As, Cr, etc. are adsorbed onto MPs/NPs [143,144]. MPs like PET and polycarbonate (in the form of fibres and granules) prevail enormously on dust particles present in polluted urban cities (Fig. 1c) [145,146]. Mercury conjugates with MPs, which in turn influences the bioaccumulation of mercury more inside tissues, as seen in *Dicentrarchus labrax* juveniles. It makes them exhibit more toxic properties like neurotoxicity, oxidative stress etc. (Fig. 1b) [147]. Moreover, humans are exposed to a variety of environmental contaminants, requiring research on the combined effects of plasticizers like BPA, PBDE, and phthalates with other chemicals to understand potential synergistic or antagonistic interactions.

## 6 MPs/NPs and associated plasticizers affecting the hypothalamus-pituitary axis (HPA)

### 6.1 Importance of the hypothalamus and pituitary gland

The hypothalamus and pituitary make up the major endocrine organs that connect and regulate various physiological processes of the body. The hypothalamus is a small section of the brain located at the base, connecting the nervous and endocrine systems. The pituitary, on the other hand, is a bi-lobed structure that remains attached just below the hypothalamus through a stalk called the infundibulum. The pituitary is located in a highly protected area in the depression of the sphenoid bone called the sella turcica [149]. The hypothalamus and pituitary despite being small organs, orchestrate complex harmony of hormonal messages. The pituitary has an anterior and a posterior part, both having different functions, and releasing different hormones into the bloodstream. The posterior pituitary produces no hormones by itself, but stores two hormones produced by the hypothalamus, namely oxytocin and antidiuretic hormone (ADH), and releases them when required. On the contrary, the anterior pituitary releases or stops hormone production under the influence of the tropic hormones released from the hypothalamus. The hypothalamus secretes certain hormones like growth hormone-releasing hormone (GHRH), thyrotropin-releasing hormone (TRH), corticotrophin releasing hormone (CRH), gonadotropin releasing hormone (GnRH) and prolactin releasing hormone (PRH). These influence the G-protein coupled receptors of the anterior pituitary to signal the release of growth hormone (GH), thyroid stimulating hormone (TSH), adrenocorticotropic hormone (ACTH), follicle stimulating hormone (FSH), luteinizing hormone (LH) and prolactin. The hypothalamus also produces growth hormone inhibiting hormone (GHIH) and prolactin inhibiting hormone (PIH) [46]. All these hormones play crucial roles in the maintenance of overall human health and well-being [150]. For example, growth hormone stimulates the breakdown of fat to make energy. During strenuous exercise, the hypothalamus releases GHRH, prompting the anterior pituitary to produce GH to generate ATP [151]. Similarly, ACTH influences the adrenal gland to produce cortisol, which controls stress responses [152]. Thyroid stimulating hormone (TSH) has a major role in metabolism, FSH/LH affect the testes and ovaries, and prolactin helps in lactation. The bodily functions are heavily dependent on the levels of these hormones and their feedback mechanisms used to regulate their increase or decrease. Oxytocin is regulated by positive feedback, which means that more oxytocin release triggers more oxytocin production [153].

### 6.2 EDCs affecting various hormones

MPs and NPs enter the bloodstream through various ingestion and inhalation processes, eventually targeting and affecting the hypothalamus and pituitary glands. Reports indicate behavioural abnormalities resulting from exposure to MPs and NPs, which can suppress acetylcholinesterase function and alter neurotransmitter levels. In mice exposed to BPA, researchers observed astrocyte activation and a range of inflammatory responses in the hypothalamus due to the activation of the toll-like receptor [TLR4], which is essential for inflammatory responses in the central nervous system [47]. The hormone kisspeptin, which is essential for zebrafish reproduction, has resulted to get decreased by exposure to MPs [59]. In the male offspring of rodents, BPA increases the number of Kiss1 neurons in the anteroventral periventricular nucleus (AVPV), while in the female offspring, it increases the number of Kiss1 cells in the rostral periventricular region of the third ventricle [48]. Neuroendocrine disturbance and reduced hypothalamic neuronal activity are caused by plastic chemicals like BPA and BPS. Phthalates alter GnRH levels by interacting with the genes of G protein-coupled receptors [GPCRs] on pituitary cells and alter FSH and LH ratio by interfering with their receptors on Leydig cells as a result of HPG axis disruption. These effects disrupt the normal function of steroidogenic enzymes and steroid hormones [60].

### 6.3 Effects on humans

Numerous studies have demonstrated the detrimental effects of MPs/NPs on the signalling pathways of the hypothalamus-pituitary axis through experiments on animal models, as discussed above. The HPA includes an interconnected network comprising the thyroid, adrenal, testicular, and ovarian axes. Any impact on the hypothalamus and pituitary consequently disrupts the functioning of these organ systems. The hypothalamus secretes hormones that regulate various pituitary activities, including growth, lactation, metabolism, and milk supply [48]. EDCs carried by attachment to MP and NP surfaces affect the pituitary gland by activating pituitary hormones such as prolactin and TSH. These interactions can induce the formation of prolactinoma, a non-cancerous tumour of the pituitary gland [49]. The effects on the hypothalamus-pituitary-gonadal (HPG) axis result in delayed formation of secondary sexual characteristics, male and female infertility, and stunted fetal development. The effect of plasticizers on the hypothalamus-pituitary-thyroid (HPT) axis causes the thyroid stimulating hormone levels to get significantly altered. This affects the homeostasis and the glucose levels required for the body's normal functioning [50]. An effect on the hypothalamus-pituitary-adrenal axis lowers the weight of the adrenal gland and affects the hormonal secretions from the cortex and medulla regions of the gland [154].

## 7 MPs/NPs and associated plasticizers as thyroid disrupting chemicals (TDC)

### 7.1 Thyroid gland and its importance

The thyroid gland, a small, butterfly-shaped organ located near the throat, produces hormones that regulate the body's growth, development, and metabolic rate. It plays a crucial role in brain development, calcium regulation, cardiac health, muscle function, and digestive processes. The gland secretes three main hormones: T4 (3,5,3′,5′-tetraiodo-l-thyronine), T3 (3,5,3′-tri-iodo-l-thyronine), and Calcitonin. Thyrotrophs in the anterior pituitary gland secrete thyroid-stimulating hormone (TSH) under the influence of thyroid-releasing hormone, which is secreted by hypothalamic neurons regulated by the hypothalamic-pituitary axis. TSH stimulates thyroid follicular cells to release T4 and T3. T4 functions primarily as a pro-hormone, circulating in the serum and converting to T3 at the tissue level through an enzymatic process that removes the 5′-iodine atom from T4 via local deiodinases. Low levels of T3 and T4 stimulate TSH release, while high levels of T3 and T4 inhibit it, demonstrating a negative feedback mechanism on TSH levels [[155], [156], [157]]. Notably, under normal circumstances, the thyroid generates and releases the majority of thyroid hormone (TH) as T4. Steady-state concentrations of T4 in the serum are significantly higher than those of T3 [[158], [159], [160]]. Since T4 gets converted into the hormone T3, T4 in their free state in the serum is a significant indicator used to measure hypothyroidism or hyperthyroidism [155]. Increased wakefulness, alertness, development of centres responsible for fetal growth, stimulation of bone remodelling and response to environmental cues are the results of T3's stimulation [161]. Therefore, interference with thyroid homeostasis can be harmful and have an impact on the body's general state of health.

### 7.2 Interactions affecting signalling pathways

Thyroid disrupting chemicals (TDCs) are foreign compounds that interfere with the production, secretion, binding mechanism, transport, or action of various thyroid hormones. Prolonged exposure to plastic particles and related compounds depletes thyroid endocrine function, reducing the hormone's ability to control metabolism, growth, reproduction, and development [87]. The industrial chemicals, also used as plasticizers in MPs/NPs, have been identified to hinder the normal physiological functioning of the hypothalamus-pituitary-thyroid (HPT) axis. These include Polybrominated Diphenyl Ethers (PBDEs), Bisphenol-A Polychlorinated Biphenyls (PCBs), Perchlorate, and Phthalates [162]. Thus, these plasticizers act as TDCs, which can also enter the human body by associating themselves with persistent organic pollutants (POPs). These TDCs enter the body through the digestive tract and deregulate T4 and T3 biochemical pathways.

Animal studies have identified several mechanisms by which PCBs may affect the thyroid gland. These mechanisms include increased biliary thyroid hormone (TH) excretion, interference with thyroid hormones binding to transthyretin (TTR)—the main TH-binding protein in rodents—disruption of the pituitary-thyroid axis, interaction with T3 receptors, and alterations in deiodinase activities. Each of these pathways contributes to the overall impact of PCBs on thyroid function [[163], [164], [165], [166]]. A study has shown that PCBs have a positive correlation with T3 and reverse T3 found in maternal serum during gestation, thus indicating a negative influence on the deiodinase type 3 activity [167].

**Fig. 2:**
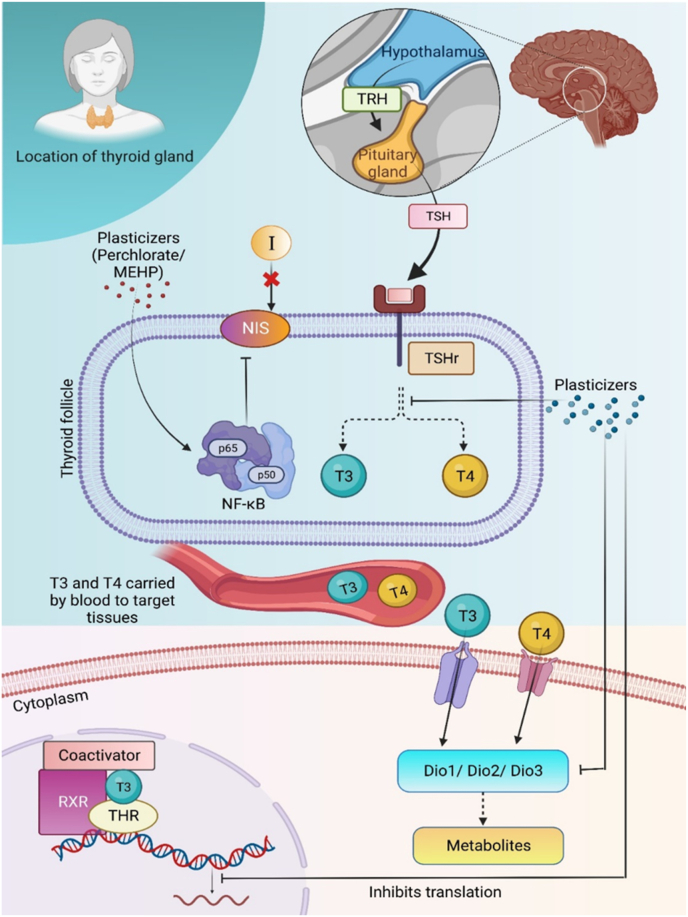
Effect of plasticizers associated with MPs/NPs on the thyroid gland. Thyrotropin-releasing hormone (TRH) is released from the hypothalamus, signalling the pituitary gland to release thyroid-stimulating hormone (TSH). TSH binds to its receptor (TSHr) at the follicle of the thyroid gland, initiating the synthesis of thyroid hormones T3 and T4. These hormones are transported through the bloodstream to target tissues, where they enter the cytoplasm via membrane transporters. Within the cytoplasm, they regulate the synthesis of metabolites and transcriptional activities. Plasticizers found in MPs/NPs, such as perchlorate, mono(2-ethylhexyl) phthalate (MEHP), and di(2-ethylhexyl) phthalate (DEHP), downregulate sodium-iodide symporter (NIS) expression by activating the NF-κB pathway, consequently impairing iodine uptake [83,84]. Plasticizers like PCB, phthalates, etc. interfere with thyroid hormone receptors, and cause disruption in the thyroid hormone related gene expression activity (e.g. Dio1/dio2 gene expression) [79,171].

Plasticizers like phthalates cause disruption in the thyroid hormone related gene expression activity, as seen in the fish - Japanese medaka. Phthalates interfere with the *dio2* gene expression, thus affecting the inflation of the swim bladder to prevent the fish from having a normal swimming movement [79]. From studies conducted on marine organisms like zebrafish, it was seen that the plasticizer mono-(2-ethylhexyl) phthalate (MEHP) decreased level of T4, increased T3, and affected the genes involved in HPT axis like *nis, NK2 homeobox 1 (nkx2.1), pared box 8 (pax8), thyroglobulin (tg), dio1, ttr, and dio2* [80]. By preventing T3 from attaching to its receptor and decreasing transcriptional activity mediated by thyroid hormone receptors, BPA can interfere with thyroid hormone action and affect thyroid functioning [66]. PBDEs are known to lower the levels of thyroid hormones in the blood by altering T4 binding and decreasing the quantity of T4 in serum. The thyroid stimulating hormone subunit and deiodinase type 2 (deio2) are two examples of the many genes whose transcriptions are altered by PBDEs, therefore disrupting the HPT axis in this way [61,62]. The sodium-iodine symporter (NIS) is an integral membrane glycoprotein located on the membrane of thyroid follicular cells and breast cells, and it regulates iodine uptake, which is crucial for efficient thyroid hormone synthesis. Perchlorate inhibits this glycoprotein. Additionally, plasticizers like MEHP and DEHP activate the NF-κB pathway, a canonical signalling pathway that induces rapid and transient transcription to regulate various proinflammatory genes and mediate inflammatory responses. Activation of this pathway leads to the down-regulation of NIS, impairing thyroid iodine uptake and consequently disrupting the gland's normal function [83,84]. (Fig. 2). Research has demonstrated that exposure of rats to polystyrene nanopolymers (PS-NPs) at doses of 1, 3, 6, and 10 mg/kg/day for five weeks suppresses the production of T3, FT3, and FT4, as well as the levels of thyroid hormones in the bloodstream [72]. In mammals, PBDEs undergo biotransformation through oxidative metabolism, resulting in the formation of hydroxylated polybrominated diphenyl ethers (OH-BDEs) and bromophenols [168,169]. OH-BDEs exhibit a closer structural resemblance to endogenous thyroid hormones, which suggests that they might be accountable for some of the observed toxicity associated with PBDEs [170].

### 7.3 Impact on humans

Humans can be exposed to plasticizers through various routes, including oral ingestion, inhalation, and dermal contact. Polychlorinated biphenyls (PCBs) have been observed to impact maternal thyroid hormone (TH) levels during pregnancy, consequently influencing fetal TH homeostasis due to their interdependence. If maternal TH levels are diminished, fetal TH levels may also decrease, potentially affecting brain growth and development. Elevated levels of thyroid-stimulating hormone (TSH) in newborns are indicative of reduced thyroid function, reflecting the adverse impact of low thyroid levels during fetal development [50]. Lower TH levels have a profoundly negative impact on the neural development of the fetus, affecting its normal physiology in the process. PCBs also pose a threat to maternal milk since a higher percentage content in the milk has shown to have detrimental effects on total T4 and total maternal T3. The study found that milk contained an average of 74.86 pg of total PCB-dioxin toxic equivalent (TEQ) per gram of fat, planar-PCB was 19.95 pg TEQ per gram of fat, and nonplanar-PCB was 22.75 pg TEQ per gram of fat [172]. A study investigated 29 children exposed to high doses of the plasticizer Di-(2-ethylhexyl) phthalate (DEHP), 23 children exposed to low doses, and 8 children with no DEHP exposure. Results revealed significantly lower blood levels of thyroid-stimulating hormone (TSH) in both the high- and low-dosage groups. Intriguingly, a 6-month follow-up study showed no alteration in TSH levels, but significant changes in serum triiodothyronine (T3) levels were observed in the high-dose group (n = 13). These findings suggest that exposure to high doses of phthalate-contaminated meals may lead to significant alterations in serum TSH levels in children or humans [51]. Phthalate metabolites were found in urine samples of children aged 4–9 years with the highest monobenzyl phthalate content [81]. A negative connection between metabolites and the serum levels of FT3 and TT3 in females was also discovered, which further indicates that phthalate metabolites' effects on the TH system can differ depending on a person's gender. According to research, women are 5–20 times more likely than males to experience thyroid-related issues [82]. The most prevalent kind of cancer is thyroid carcinoma, and numerous studies have hypothesized that endocrine-disrupting pollutants, which also include PBDEs may unbalance the TH system leading to cancer [[52], [53], [54]]. Numerous studies have reported that PBDEs can disrupt human thyroid function by interfering with the transport of thyroid hormones [[63], [64], [65]]. Urinary perchlorate also disrupts thyroid functioning in both men and women [[173], [174], [175]].

The SCREENED project aims to develop advanced *in vitro* assays using rodent and human thyroid cells organized into three distinct three-dimensional (3D) constructs, each designed to increasingly mimic the native thyroid gland's structure and function. These constructs include a 3D organoid from stem cell-derived thyrocytes, a decellularized thyroid lobe repopulated with stem cell-derived thyrocytes, and a bioprinted organoid mimicking the spatial and geometrical features of a native thyroid. Housed in a modular microbioreactor with innovative sensing technology, these constructs will utilize superparamagnetic biocompatible particles to create “magnetic cells” for precise cell positioning. The 3D assays will screen endocrine disruptors (EDs) for thyroid function effects in a sex-specific manner, providing higher sensitivity and specificity than traditional 2D assays and animal models. Proteogenomic analysis and computational modelling will support a mechanistic understanding of the adverse effects of EDs on thyroid function [176]. The successful completion of the project may yield significant insights, shedding light on various research gaps about the mechanistic effects of EDCs on the thyroid organ.

## 8 Disruption of the adrenal gland by MPs/NPs and associated plasticizers

### 8.1 Adrenal gland and its importance

**Fig. 3:**
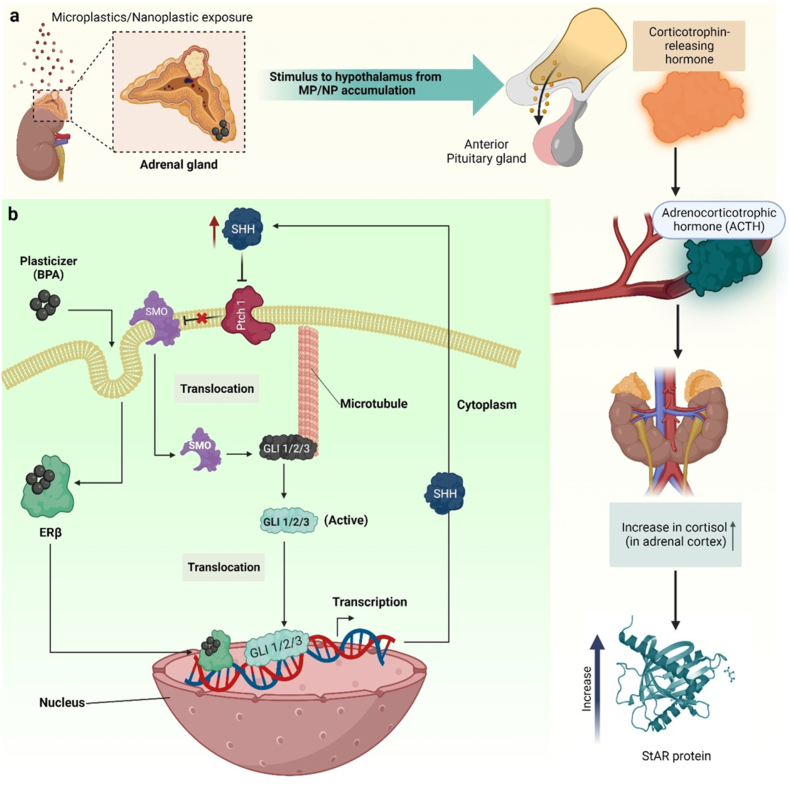
Effect of MPs/NPs and associated plasticizers on the adrenal gland. (a) Exposure of the adrenal gland to polystyrene nanopolymers (PS-NPs) and plasticizer (BPA) activates the Hypothalamic-Pituitary-Adrenal (HPA) stress axis. This stimulates release of ACTH from pituitary gland, which in turn increases the level of cortisol in the bloodstream. BPA increases the plasma corticosterone level which in turn increases StAR protein via an unidentified mechanism [73,68]. (b) The possible chemical mechanism via which BPA increases cell proliferation in human adrenal cells has been depicted schematically.

The adrenal gland, comprising the adrenal cortex and adrenal medulla, serves as a pivotal endocrine organ responsible for secreting hormones such as cortisol, aldosterone, epinephrine, and norepinephrine. Positioned atop each kidney, these small, triangular-shaped glands, also known as suprarenal glands, play a vital role in regulating physiological processes. The hypothalamus, pituitary gland, and adrenal glands collectively form the hypothalamic-pituitary-adrenal (HPAd) axis, a complex network characterized by direct impacts and feedback loops. Factors such as stress, disease, blood cortisol levels, and circadian rhythm influence the secretion of corticotropin-releasing hormone (CRH) from the hypothalamus. While glucocorticoids fulfil essential functions, including stress response regulation, excessive levels can prove detrimental. Prolonged exposure to high glucocorticoid concentrations is implicated in hippocampal atrophy observed in both humans and animals experiencing severe stress. Hippocampal impairments may constrain memory resources crucial for developing appropriate stress responses. Remarkably, the healthy adult adrenal glands express approximately 70 % of the 20,000 protein-coding genes in the human genome, highlighting their significant genomic activity [177]. Members of the cytochrome P450 superfamily of enzymes are among those genes specific to the adrenal glands that are expressed at the greatest levels [178]. A paradigm that integrates physiologic and pathologic processes has been developed as a result of the discovery of the molecular pathogenesis of several hereditary illnesses with adrenocortical symptoms. Both embryonic development and homeostasis depend heavily on the insulin-like growth factor (IGF), protein kinase A (PKA), hedgehog (HH), and Wnt signalling pathways. In addition, both normal and abnormal adrenocortical functions depend on telomere maintenance and protection. Shh is the only ligand found in the adrenal gland. When Shh-expressing cells communicate with non-steroidogenic (Sf1-negative) cells implanted in the adrenal capsule, Shh signalling is triggered. These Shh-responsive cells have a cell-surface receptor called patched homolog 1 (Ptch1), which Shh ligands bind to. The zinc finger glioma-associated oncogene family (Gli) of transcription factors is activated downstream by Shh binding, which relieves the Ptch1-mediated control of the signal transducer smoothened homolog (Smo) (Fig. 3b). The normal physiological functions like stress factors, blood pressure and other metabolisms are controlled by the hormones secreted by adrenal glands and thus any kind of discrepancy is harmful to the body's homeostasis.

### 8.2 EDCs affecting signalling pathways

The plasticizers from various kinds of MPs/NPs acting as EDCs in the environment, interfere with the adrenal signalling pathways and block the production of the required hormone or its precursors. PS-NPs when administered to zebrafish, modify glucose homeostasis by boosting cortisol secretion (Fig. 3a). This shift in cortisol levels impacts key molecules like neurotransmitters, enzymes, and receptors, by hindering the brain's electrical impulses, which in turn affects behavior [73]. By raising dose-dependent levels of the steroidogenic acute regulatory protein (StAR) in the offsprings of mice, prenatal exposure to BPA has been demonstrated to result in higher plasma corticosterone levels. It has been demonstrated that BPA increases StAR protein levels via an unidentified mechanism, which calls for research to be conducted on it [179] (Fig. 3a). It also alters signalling via endoplasmic reticulum and Sonic Hedgehog [SHH] signalling, activating cyclin D1 and cyclin D2, and changing the proliferation of adrenal cells. BPA easily crosses the cell membrane and enters the cytoplasm, where it binds to and activates the ER. The active ER translocate to the nucleus, where it increases Shh gene transcription, resulting in increased production of Shh mRNA and protein. Shh is released, works in autocrine/paracrine mode, and binds to the Patched 1 (Ptch1) receptor, blocking Ptch1 from inhibiting smoothened (SMO). SMO is released into the cytoplasm, resulting in the release of a complex formed by the Shh transcription factor Gli1. In the nucleus, Gli1 undergoes translocation and interacts with the promoters of pivotal proliferation proteins, namely CCND1 and CCND2, thereby enhancing transcriptional activity and consequently promoting cell proliferation [55]. (Fig. 3b). A study conducted on pregnant mice aimed to evaluate whether BPA disrupts signalling mechanisms, thereby influencing adrenal steroidogenesis in offspring. The findings revealed that BPA exposure increased adrenal gland weight in both male and female offspring. Additionally, BPA led to elevated plasma corticosterone levels in both sexes. However, specifically in female offspring, BPA induced the expression of two crucial factors, StAR and cyp11A1, responsible for regulating the steroidogenic pathway within the adrenal glands. Notably, BPA did not impact plasma adrenocorticotropic hormone (ACTH) levels or the expression of SF-1, the primary steroidogenic transcription factor, in the adrenal glands of either sex [68]. DEHP, an indispensable plasticizer, is linked to decreased angiotensin II expression in adult adrenal glands, which lowers levels of aldosterone [56]. Exposure to PBDEs, such as 4-bromodiphenyl ether (BDE3), elevates serum levels of aldosterone and corticosterone. In rats exposed to 200 mg/kg of BDE3, upregulation of Cyp11b1 expression was observed alongside disruption of AMP-activated protein kinase (AMPK) signalling, evidenced by a reduction in its phosphorylation [57]. Octyl phenol and nonyl phenols, which are made from ethoxylates, function as an EDC and harm the adrenal gland's endogenous estrogenic cascade. A study conducted on the vertebrate *Podarcis sicula* lizard revealed that nonyl phenol, upon attaching itself to the estrogen receptor (ER), operates as an estrogenic substance. This leads to the replication of the effects induced by estradiol 17β (E2), resulting in prolonged activation of hypothalamic corticotropin-releasing factor (CRF) production. Consequently, the absence of the negative feedback loop of the hypothalamic-pituitary-adrenal (HPA) axis disrupts systemic reactions in the organism [86].

### 8.3 Effect on humans

The effect of MPs/NPs and associated plasticizers as endocrine disruptors has been widely investigated in in vivo models as discussed above, but their precise mechanisms of attacking the human system require further exploration. However, a few significant studies have shed light on the potential pathways involved. BPA as an endocrine disruptor has been shown to be contained in more concentration in the serum of patients with nonfunctional adrenal incidentaloma as compared to the controls [69]. Organophosphates, such as isopropylated triphenyl phosphate [IPTPP], are known to augment the relative weight of the adrenal gland by causing hypertrophy of the adrenal cortex in the zona fasciculata region [58]. A study investigated the impact of organophosphate esters (OPEs) on H295R human adrenal cell phenotypic endpoints and function, shedding light on a previously understudied area. Results reveal that most OPEs alter oxidative stress, mitochondrial dynamics, lysosomal content, and lipid droplet formation. Triaryl-OPEs and one nontriaryl OPE, particularly isopropylated triphenyl phosphate (IPPP) and tris(methylphenyl) phosphate (TMPP), demonstrate greater potency than the legacy brominated flame retardant, BDE-47. Furthermore, OPE exposure affects steroidogenic activity in adrenal cells, with distinct alterations in cortisol and aldosterone production and StAR expression patterns, highlighting the adrenal gland as a significant target for these endocrine-disrupting chemicals [180]. Similarly, exposure of DEHP and MEHP to H295R cells resulted in upregulation of 17β-HSD1 and CYP19A1 and downregulation of CYP17A1, CYP11A1 and StAR [181]. Dichlorodiphenyltrichloroethane (DDT) causes abnormalities in the cortex and medulla of the adrenal gland and can bio-accumulate in the thymus, brain, and even adipose tissue. It also disrupts hormonal secretion in cortical and chromaffin cells and inhibits the formation of thyroxine hydroxylase in chromaffin cells [182].

## 9 MPs/NPs and associated plasticizers affecting the reproductive system

The reproductive system and its associated disorders constitute a significant concern within the medical field, prompting ongoing research efforts. Due to their minute size, micropolymers and nanopolymers possess the capacity to infiltrate the reproductive cells, tissues, and organs of organisms, thereby perturbing normal functioning, morphology, and physiology in both male and female reproductive systems. Plasticizers present in the environment can potentially interact with endocrine disruptors, thereby disrupting the body's equilibrium and impeding developmental phases such as social behaviour, pregnancy, puberty, and sexual dimorphism [183]. Plasticizers can trigger responses in the neuroendocrine system as they are hormonally active compounds, which in turn can affect the reproductive health of the organism. With the increase in exposure to polystyrene microplastic and nanoplastic, the accumulation of MPs/NPs in the gonads can increase because of the dysfunctional intestinal barrier [184]. Although the primary mechanism of the uptake of MPs and NPs from the primary site remains unclear, phagocytosis and endocytosis have been established to be prevalent at the cellular level. The effects of MPs/NPs affecting the female and male reproductive system are discussed as follows.

### 9.1 Female reproductive system

The mammalian female reproductive system encompasses several vital components, including the ovaries, fallopian tubes, cervix, and vagina. Within the ovaries, granulosa cells play a pivotal role in secreting essential female reproductive hormones, such as estrogen, progesterone, and other associated hormones. Micro and nanopolymers (MPs/NPs) hold the potential to infiltrate ovarian granulosa cells, thereby augmenting the number of ovarian follicles while significantly diminishing anti-Müllerian hormone levels. This phenomenon instigates oxidative stress, ultimately precipitating cell apoptosis and contributing to uterine and ovarian fibrosis [74,75]. Exposure to endocrine disruptors such as Di-2-ethylhexyl phthalate (DEHP) can impact reproductive tract development, leading to reduced birth weight and a significant decrease in anogenital distance in female offspring [85]. Plasticizer like BPA bears a strong affinity for the estrogen receptors, Erα and Erβ, since it exhibit estrogen mimicking behaviour by the removal of the phenol group [70]. Decreased motivation for reproduction was observed when planktonic crustacean *Daphnia magna* were exposed to increased levels of plastic particles, because of increased inter-brood periods and decreased average blood production [88]. Wu et al. elucidated that MPs/NPs have the potential to initiate the TR4/NOX2 signalling axis, leading to oxidative stress. This oxidative stress, in turn, activates the Notch and TGF-β signalling pathways, ultimately culminating in uterine fibrosis [75].

**Fig. 4:**
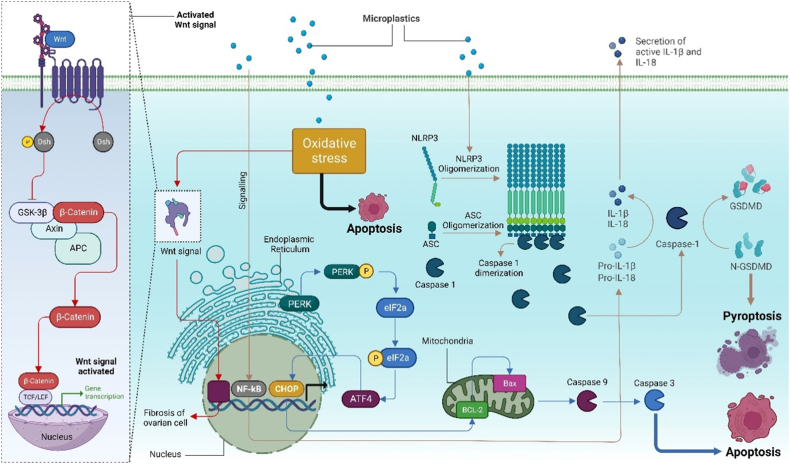
Micropolymers and associated plasticizers activating various pathways like Wnt/β-catenin signalling and NLRP3/caspase-1 pathway. PS-MPs induce oxidative stress leading to β-catenin release. In the nucleus, it binds to TCF/LEF, thus activating the signalling pathway [90]. PS-MPs also lead to pyroptosis and apoptosis of granulosa cells by activating the NLRP3/caspase-1 pathway through oxidative stress [185].

PS-MP induces granulosa cell death through apoptosis and proptosis by activating the NLRP3/caspase pathway via induced oxidative stress (Fig. 4). Stimulation by PS-NPs leads to the translocation of NF-κB subunits to the nucleus, enhancing the transcription of the pro-IL-1β gene and facilitating NLRP3 assembly. Activated caspase-1 dimers then process pro-IL-1β and pro-IL-18 into their biologically active forms, ultimately triggering pyroptosis in granulosa cells. Phosphorylation of PERK in the endoplasmic reticulum results in eIF2a phosphorylation, subsequently promoting ATF4 transcription and CHOP gene expression. Bcl-2 in the mitochondria converts to Bax, releasing caspase-9 and caspase-3, thereby inducing granulosa cell apoptosis. Concurrently, activation of the Wnt/β-Catenin signalling pathway exacerbates fibrosis in ovarian cells. PS-MP-induced oxidative stress enhances Wnt ligand availability, leading to the phosphorylation of β-catenin by CK1 kinases and GSK3β in the cytoplasm. The binding of axin to LRP5/6 allows β-catenin degradation complex dissociation. The liberated β-catenin translocates to the nucleus, facilitated by its binding partner FOXM1. In the nucleus, β-catenin binds to TCF/LEF, releasing the Groucho/TLE repressor and activating the signalling pathway [89,90] (Fig. 4). Hou et al., showed that there was an increase in the level of malondialdehyde (MDA), while the activity of glutathione peroxidase (GSH-Px), superoxide dismutase(SOD), and catalase (CAT) were decreased in the ovarian tissue [90,185].

A study assessed the impact of exposure to nine organophosphate esters (OPEs) on reproductive cells, including KGN human granulosa cells. Several OPEs increased the number of mitochondria, decreased lysosomes, enlarged the total area of lipid droplets, and induced oxidative stress in KGN cells [186]. In a study by Amar et al., bisphenol S was administered to granulosa cell cultures, resulting in a dose-dependent decrease in progesterone and estradiol secretion. Furthermore, exposure for 48 h led to a reduction in CYP11A1 protein expression levels and an increase in ERRγ expression [187]. These changes highlight the heightened sensitivity of human granulosa cells to OPE exposure, underscoring the potential reproductive toxicity of these chemicals.

### 9.2 Male reproductive system

**Fig. 5:**
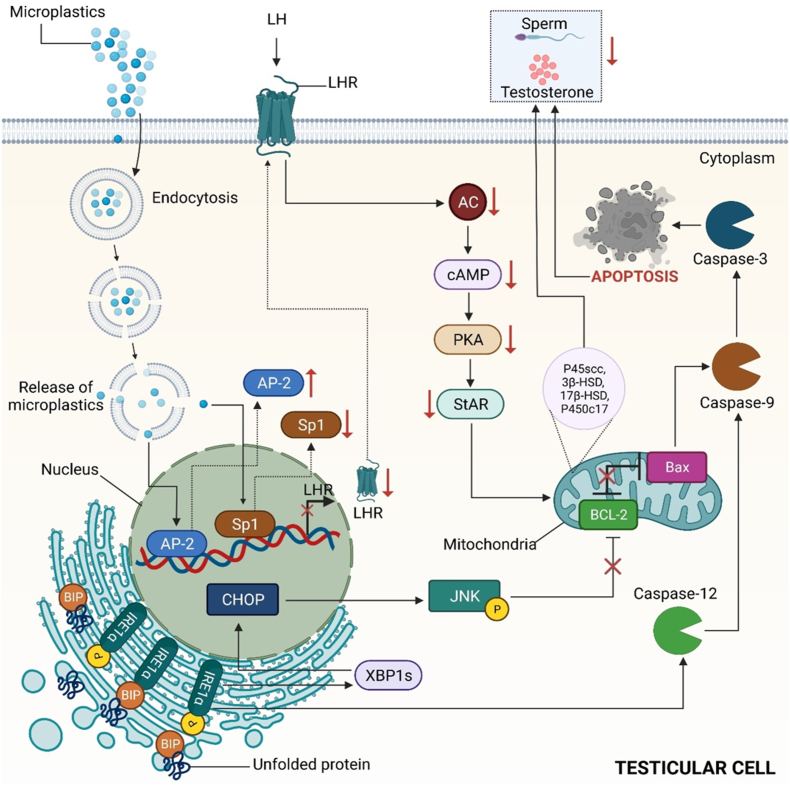
Effect of micropolymers on the male reproductive system. MPs lead to the downregulation of LH-mediated LHR/cAMP/PKA/StAR pathway, bearing the responsibility for the decreased level of testosterone.

The male reproductive system comprises several sex organs crucial for human reproduction, located externally in the pelvic region. The development, maturation, and establishment of reproductive organs in the male system occur during embryonic and perinatal stages. Morphological abnormalities, low sperm count, and the absence of spermatozoa in semen are among the key factors contributing to male infertility [188]. Plasticizers associated with MPs/NPs like phenols, phthalates, BPA, DEHP and their metabolites are among the endocrine disruptors which can cause male infertility [71]. With an increase in exposure to DEHP, there is delayed formation of secondary sexual characteristics and often results in abnormalities. Along with a reduced anogenital distance, an incomplete testicular descent is also observed [71]. In a study, the potential toxicity of polystyrene MPs was assessed in male mice's reproductive system, resulting in abscised and disorderly arranged spermatogenic cells and multinucleated gonocytes in the seminiferous tubule. On accumulation of microplastic in the body, alteration in the testicular shape and decrease in serum testosterone, Luteinizing hormone (LH), and Follicle Stimulating hormone (FSH) levels were documented [91]. The downregulation of the LH-mediated LHR/cAMP/PKA/StAR pathway bears the responsibility for the decrease in the level of testosterone [189]. In a study by Jin et al., Leydig cells were found to internalize microparticles (MPs) through endocytosis. This internalization resulted in the downregulation of key components of the LH hormone receptor (LHR), steroidogenic enzymes, and StAR, achieved by inhibiting the activation of the cAMP/AC/PKA pathway. Consequently, the release of caspase 3 and caspase 9 was observed, triggering apoptosis and leading to a reduction in testosterone levels. Additionally, the study revealed that the transcription factor Sp1 was downregulated while the suppressor AP-2 was upregulated (Fig. 5). Notably, the accumulation of nanoparticles (NPs) in the body has been demonstrated to adversely affect the reproductive system of earthworms, resulting in deformities in sperm cells. This ultimately damages the plasma membrane, reduces sperm density and viability, and diminishes the production of mature sperm bundles [76].

DEHP, a widely used plasticizer, is commonly present as an additive on the surface of nanoparticles (NPs), contributing to endocrine disruption. In a study by Li et al., C57BL/6J mice were administered polystyrene nanoparticles, DEHP, and DEHP-conjugated PS-NPs to investigate the adverse effects of DEHP on the male reproductive system. While low levels of polystyrene NPs did not significantly alter sperm quality, both DEHP and the conjugate led to decreased sperm quality and affected the structure of the testis and epididymis. Furthermore, exposure resulted in a slight decrease in testosterone levels, and PS-NPs exacerbated the reduction of ZO-1 induced by DEHP, potentially leading to tight junction damage in the testis [77]. In a separate investigation, researchers examined the impact of nanopolystyrene beads on the spermatozoa of *Crassostrea gigas* (oysters) to elucidate the effects of nanoplastics on marine organisms. Given the oysters' external fertilization process, nanopolystyrene beads were exposed to the spermatozoa for a duration of 1 h. Results revealed the adhesion of plastic particles to the external surface of the spermatozoa, with increasing nanoplastic concentrations correlating with heightened spermiotoxicity, evidenced by reduced motility and velocity, consequently diminishing embryogenesis success. Disruption of homeostasis likely underlies the disturbances in reproductive function, ultimately affecting reproductive success [78].

Additional research is needed to understand the mechanism of reproductive failure caused by the presence of MP and NP in the reproductive organ, the prevention of which would provide improved healthcare facilities.

## 10 Research gaps and future perspectives

Assessing the exact global occurrence percentage of MPs/NPs across terrestrial and aquatic environments remains a daunting challenge due to their escalating levels. The proliferation of these plastic particles has precipitated a plethora of adverse impacts on biotic health. While considerable attention has been devoted to studying the effects of MPs/NPs on endocrine glands, there exists a notable discrepancy in the emphasis placed on each gland. For instance, extensive research has targeted the disruption of signalling pathways in the thyroid and reproductive glands compared to others. A more mechanistic approach is warranted to elucidate the dissociation of plasticizers from MPs/NPs and their subsequent binding to endocrine-disrupting chemicals (EDCs) in the environment. Such investigations are crucial for devising strategies to mitigate the formation of these harmful conjugates in the future [190]. To date, significant focus has been directed towards understanding how different plasticizers impact the signalling pathways of the thyroid gland and gonads. However, research on the effects of MPs/NPs on the female reproductive system lags behind that of the male. Despite its importance as an endocrine organ, the adrenal gland's response to EDCs has primarily been explored in *in vivo* animal models, elucidating the affected signalling pathways. Conversely, scant information exists regarding the effects of MPs/NPs or associated plasticizers on the human adrenal gland. The experiments on the effects of micropolymers (MP) and nanopolymers (NP) on the function of human endocrine system were usually carried out using the animal models including zebra fish, mice and rats, the organs of these models are quite different from human being. Recently, the organoids of human endocrine organs which were self-organized from the human embryonic stem cells or human induced pluripotent stem cells have been used to test the developmental toxicity and endocrine disruption. It successfully resolved the issue of species differences between human being and the animal models. Furthermore, to our knowledge, the impact of MPs/NPs on other endocrine glands with lesser prominence, such as the pineal and parathyroid glands, remains largely unexplored in research efforts.

## 11 Conclusion

Our society's pervasive reliance on plastic has led to a concerning global pollution issue with microplastics (MPs) and nanoplastics (NPs) degradation. Despite limited attention, the issue of ingested MPs/NPs bioaccumulation in mammalian tissues and organs is emerging as a significant concern, given its potential to induce a range of adverse health effects, including endocrine abnormalities, reproductive toxicity, gut microbiota dysbiosis, and impaired immune responses. As both agonists and antagonists for various hormone receptors, diverse endocrine-disrupting chemicals (EDCs) in the form of MPs/NPs or associated plasticizers can readily infiltrate the body, precipitating endocrine toxicity. Although conclusive research findings regarding the direct impacts of MPs and NPs on key endocrine glands such as the hypothalamus, pituitary, and adrenal gland remain elusive, their potential disruption of the hypothalamic-pituitary axis poses a significant threat to human physiology. Disruption of this axis compromises interconnected signalling pathways vital for regulating numerous physiological processes, necessitating immediate preventive measures against EDC-induced perturbations. A comprehensive understanding of how EDCs like MPs/NPs influence specific endocrine signalling systems is imperative. Identifying the binding sites and carriers of such EDCs within the endocrine system holds promise for the development of targeted interventions aimed at eradicating these pollutants. Therefore, concerted research efforts are warranted to ascertain the potential risks posed by MPs/NPs and their associated plasticizers, facilitating the establishment of regulations aimed at mitigating exposure to these minuscule plastic particles. Such regulatory measures are essential to safeguard public health and mitigate the deleterious effects of plastic pollution on human well-being and the environment.

## CRediT authorship contribution statement

**Utsa Saha:** Writing – review & editing, Writing – original draft, Visualization, Validation, Methodology, Investigation, Formal analysis, Data curation, Conceptualization. **Puja Kumari:** Writing – review & editing, Writing – original draft, Visualization, Methodology, Formal analysis, Data curation, Conceptualization. **Aishee Ghosh:** Writing – review & editing, Writing – original draft, Validation, Methodology, Investigation, Formal analysis, Data curation, Conceptualization. **Adrija Sinha:** Writing – review & editing, Writing – original draft, Formal analysis, Data curation, Conceptualization. **Snehashmita Jena:** Writing – review & editing, Writing – original draft, Methodology, Formal analysis, Data curation, Conceptualization. **Apoorv Kirti:** Writing – review & editing, Writing – original draft, Investigation, Data curation, Conceptualization. **Abha Gupta:** Writing – review & editing, Writing – original draft, Visualization, Formal analysis, Data curation, Conceptualization. **Anmol Choudhury:** Writing – review & editing, Writing – original draft, Visualization, Formal analysis, Data curation, Conceptualization. **Faizan Zareen Simnani:** Writing – review & editing, Writing – original draft, Visualization, Validation, Formal analysis, Data curation, Conceptualization. **Aditya Nandi:** Writing – review & editing, Writing – original draft, Visualization, Formal analysis, Data curation, Conceptualization. **Rudra Narayan Sahoo:** Writing – review & editing, Writing – original draft, Visualization, Formal analysis, Data curation, Conceptualization. **Shalini Singh:** Writing – review & editing, Visualization, Validation, Formal analysis, Data curation, Conceptualization. **Richa Mishra:** Writing – review & editing, Visualization, Validation, Software, Resources, Formal analysis. **Nagendra Kumar Kaushik:** Writing – review & editing, Visualization, Validation, Supervision, Resources, Project administration, Formal analysis, Conceptualization. **Deobrat Singh:** Writing – review & editing, Visualization, Validation, Investigation, Funding acquisition, Data curation, Conceptualization. **Mrutyunjay Suar:** Writing – review & editing, Validation, Supervision, Project administration, Funding acquisition, Formal analysis. **Suresh K. Verma:** Writing – review & editing, Writing – original draft, Visualization, Validation, Supervision, Software, Resources, Project administration, Methodology, Investigation, Funding acquisition, Formal analysis, Data curation, Conceptualization.

## Declaration of competing interest

The authors declare that they have no known competing financial interests or personal relationships that could have appeared to influence the work reported in this paper.

## Data Availability

Data will be made available on request.
